# Regulatory roles of circular RNAs in Wnt and other oncogenic signaling pathways in breast cancer progression: a comprehensive review

**DOI:** 10.1186/s40001-025-02967-9

**Published:** 2025-08-14

**Authors:** Amirreza Khalaji, Yousef Nazari, Mojtaba Pandeh, Aram Farhoudian, Leila Ghorbi, Pedram Naderi, Elham Mohebi Janagard, Samira Amin Afshari, Reza Morovatshoar

**Affiliations:** 1 Student Research Committee, Tabriz University of Medical Science, Tabriz, Iran; 2 School of Medicine, Lorestan University of Medical Sciences, Khorramabad, Iran; 3 School of Medicine, Gonabad University of Medical Sciences, Gonabad, Iran; 4 School of Medicine, Urmia University of Medical Sciences, Urmia, Iran; 5 Shiraz University of Medical Sciences, Shiraz, Iran; 6 Immunology Research Center, Tabriz University of Medical Sciences, Tabriz, Iran; 7 Endocrinology and Metabolism Research Center (EMRC), Vali-Asr Hospital, Tehran University of Medical Sciences, Tehran, Iran; 8 Clinical Research Development Center of Shahid Mohammadi Hospital, Hormozgan University of Medical Sciences, Bandar Abbas, Iran

**Keywords:** Breast cancer, CircularRNAs, circRNA biogenesis, Wnt, Wnt signaling pathway, Molecular agents

## Abstract

Circular RNAs (circRNAs) are critical regulators in breast cancer (BC) progression, particularly through their interactions with the Wnt/β-catenin signaling pathway. This comprehensive review elucidates the regulatory roles of key circRNAs, including circABCC4, circFAT1, circARL8B, and circDONSON, in modulating BC behavior. These circRNAs primarily function as microRNA sponges, influencing essential processes such as proliferation, metastasis, drug resistance, and cell survival. For instance, circDONSON promotes tumor growth and radioresistance through SOX4-mediated Wnt signaling, whereas circRNF10 exhibits tumor-suppressive properties. The dual role of circRNAs as oncogenes or tumor suppressors highlights their complexity. Their high stability and tissue-specific expression patterns position them as promising diagnostic biomarkers and therapeutic targets. Dysregulation of circRNAs modulates Wnt/β-catenin signaling, a key driver of BC progression, promoting oncogenesis and therapeutic resistance. This review synthesizes evidence from peer-reviewed literature, emphasizing the molecular mechanisms and clinical implications of circRNA–Wnt interactions. By exploring these intricate networks, we identify novel opportunities for targeted BC therapies, underscoring the potential of circRNAs to transform diagnosis and treatment. Future research should prioritize standardizing circRNA quantification and validating findings across diverse patient cohorts to enhance clinical applicability.

**Graphical Abstract:**

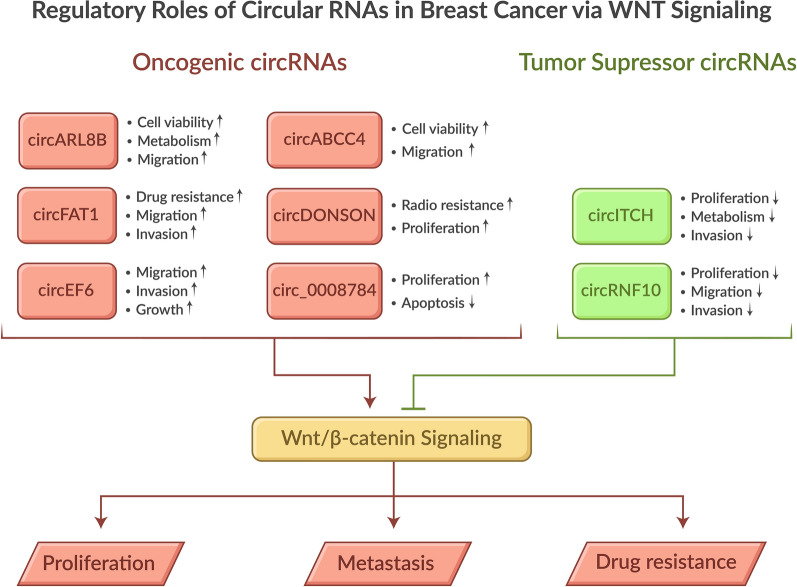

## Introduction

Breast cancers (BCs) are the second most significant risk factor for mortality among women globally and remain the leading cause of cancer-related deaths [1–3]. In 2024, the American Cancer Society projected 313,510 new cases of BCs and 42,780 related deaths in the United States, encompassing both men and women. These statistics underscore the pressing need for immediate and sustained support and resources to manage this severe disease through novel diagnostic and therapeutic approaches [4]. BCs are classified based on the expression of Human epidermal growth factor receptor 2 (HER2), progesterone, and estrogen receptors, determined through immunohistochemistry staining [5, 6]. Although historically associated with affluent nations, in 2020, countries in the poorest regions accounted for most BC-related fatalities and over half of all hospital admissions [7]. Despite medical advancements, BC incidence rates remain high, underscoring the crucial role of our understanding in informing effective interventions and empowering us to make a difference. This comprehensive review examines the intricate regulatory mechanisms of circRNAs in the Wnt signaling pathway, focusing on their molecular interactions and downstream effects in BC. We evaluate the multifaceted roles of these circRNA–Wnt pathway interactions in BC progression, emphasizing their potential implications for therapeutic development and clinical outcomes.

A significant portion of the human genome is transcribed into RNAs that do not encode proteins. These noncoding RNAs (ncRNAs) play critical roles in regulating the initiation and progression of various cancers. Based on their length, structure, and location, ncRNAs are classified into distinct types, including PIWI-interacting RNA (piRNA), microRNA (miRNA), long noncoding RNA (lncRNA), and circular RNA (circRNA), each with unique functions in cancer biology [8]. Circular RNAs (circRNAs) represent a recently characterized class of noncoding endogenous RNAs that play pivotal roles in BCs. These unique RNA molecules are generated through back-splicing of substrate mRNAs, forming single-stranded, covalently sealed RNA units [9]. CircRNAs exhibit distinctive characteristics, including stable loop structures, evolutionary conservation, and highly specific expression patterns across cell types, tissues, and developmental stages [10]. Although the precise physiological functions of most circRNAs remain primarily uncharacterized, their established roles encompass multiple mechanisms: sequestration of miRNAs and proteins, modulation of RNA polymerase II-mediated transcription, regulation of pre-mRNA splicing processes, and potential for translation [11]. Dysregulation of circRNA expression has been implicated in various malignancies, including hepatocellular carcinoma [12, 13], gastric cancer [14, 15], oral squamous cell carcinoma [15, 16], lung tumors [17], and colorectal cancers (CRCs) [18]. In BC specifically, emerging research has revealed crucial functions of circRNAs; for instance, circACAP2 promotes BC progression and growth through the regulation of COL5A1 via miR-29a/b-3p [19].

Cellular contact occurs through sophisticated extracellular signaling mechanisms, whereby cells produce specific molecular signals that bind to dedicated receptors on target cells, triggering distinct intracellular signaling cascades. This intricate network enables cellular adaptation to environmental changes [20]. Notably, recent investigations have highlighted the fundamental roles of key signaling pathways—including Notch, Hippo, and Wingless-related Integration Site (Wnt)—in various breast cancer subtypes, particularly in aggressive triple-negative breast cancers (TNBCs) [21, 22]. These pathways have emerged as promising therapeutic targets for breast cancer treatment [23]. The dysregulation of circulating circRNA levels is a significant factor in the development of BC, as it modulates the Wnt signaling pathway [24]. This pathway, particularly when dysregulated, plays a fundamental role in BC progression from initial development through metastasis [25]. The Wnt/β-catenin signaling system serves as a master regulator of critical cellular processes, including apoptosis, motility, proliferation, and tissue homeostasis [26–28]. Notably, circRNAs associated with the Wnt/β-catenin pathway demonstrate essential regulatory functions across multiple malignancies, controlling downstream targets crucial for tumor cell behavior, oncogenesis, and cancer progression [29]. The clinical significance of circRNAs extend beyond their regulatory roles, as they show promise as both diagnostic biomarkers and therapeutic targets. Their stability and tissue-specific expression patterns make them particularly attractive for clinical applications in BC management.

The Wnt pathway, extensively researched and established as one of the most crucial signaling cascades in cancer initiation and progression, is of significant importance. CircRNAs serve as key regulatory elements in this pathway. Studies have shown that Wnt signaling is upregulated across nearly all cancer types, promoting disease progression and enhancing various cancer hallmarks, including metastasis, migration, proliferation, and angiogenesis [30]. For example, circLgr4 has been shown to promote colorectal cancer invasion and carcinogenesis through Wnt/β-catenin pathway activation [31]. The Wnt signaling pathway's dysregulation represents a critical mechanism in BC development, distinct from its role in other cancers. Unlike intestinal cancers, where specific mutations drive pathway dysfunction, BCs exhibit complex patterns of Wnt signaling aberrations that interact with multiple cellular processes [32].

This review discusses evidence from an extensive analysis of peer-reviewed literature in PubMed, focusing on the complex interactions between circRNAs and the Wnt pathway in BC progression. We comprehensively examine the biochemical mechanisms and functional capabilities of Wnt pathway-associated circRNAs, but what truly sets them apart are their unique characteristics. These characteristics, which we will explore in detail, hold the key to understanding their potential therapeutic implications in BC pathogenesis.

### Overview of circular RNAs: structure, biogenesis, and biological significance

circRNAs are a class of noncoding RNAs with a covalently closed loop structure lacking 5′ caps and 3′ poly(A) tails [33]. They are primarily generated via a back-splicing mechanism from precursor mRNAs [34]. Based on their origin and structure, they are categorized into exon–intron circRNAs (EIciRNAs), exonic circRNAs (EcircRNAs), circular intronic RNAs (ciRNAs), read-through circRNAs (rt-circRNAs), and fused circRNAs (f-circRNAs) [35, 36]. Their biogenesis is regulated by RNA-binding proteins (RBPs) and intronic sequences, with additional intronic repeats, for example, Alu elements, playing a vital role in circularization [37–39]. Alternative circularization can occur through competition between RNA pairings, leading to multiple circRNAs from one gene. RBPs such as quaking (QKI), fused in Sarcoma (FUS), and Splicing factor proline/glutamine-rich (SFPQ) promote circRNA formation [40–42], whereas ADAR1 and DHX9 inhibit it by disrupting base pairing [43, 44]. Transcription factors (TFs) also influence circRNA expression by modulating host gene transcription [45]. Examples include CTCF regulating circSPARC and TFAP2C enhancing circIL4R expression [46, 47]. The recent studies have highlighted the crucial roles of circular RNAs in regulating diverse biological and pathological processes. They are involved in chromatin remodeling, transcription, translation, RNA stability, and scaffolding [48]. The following sections will discuss the diverse biological functions of circular RNAs.

#### CircRNAs modify gene activity via miRNA sequestration

The miRNA sponging effect is a thoroughly investigated function of circRNAs [10]. CircRNAs have multiple binding sites for miRNA, thereby resulting in their sponge-like function [49]. In cancers, accumulating evidence shows that circRNAs, such as circRNA-Ctfrc, act as sponges for miRNA and take part in the transcriptional regulation of target genes [50].

CDR1as, for example, sponges miR-7 and is involved in brain function and pulmonary fibrosis [51]. Circ_0025202, derived from GAPDH, functions as a tumor suppressor in HR-positive breast cancer by sponging miR-182-5p and enhancing FOXO3a expression. This process suppresses tumor cell proliferation and migration, increasing apoptosis and tamoxifen sensitivity [52].

#### CircRNAs mediate protein interactions

CircRNAs, with their ability to act as protein sponges, scaffolds, and recruiters, exert influence on a wide range of cellular and pathological processes [53, 54]. For instance, circDnmt1 promotes autophagy and tumor growth by binding to p53 and Auf1 [55]. Similarly, circPABPN1 and circFoxo3 regulate cell proliferation and senescence by sequestering HuR and other proteins [56]. Furthermore, circNSUN2 enhances HMGA2 mRNA stability, thereby promoting colorectal cancer metastasis [57].

#### Regulatory roles of CircRNAs in gene expression

Intron-derived circRNAs can enhance gene expression by engaging with chromatin reformation complexes and RNA polymerase II [35]. CircFECR1 recruits TET1 to demethylate DNA at oncogenic promoters, thereby influencing breast cancer metastasis [58]. CircEsyt2 modulates alternative splicing of p53 by regulating PCBP1, thereby impacting vascular remodeling [59]. Meanwhile, circYap suppresses translation initiation, consequently slowing breast cancer progression [60].

#### Protein-coding capacity of circular RNAs

Some circRNAs, with their unique biological functions, can serve as templates for protein production by cap-independent or cap-dependent methods. These include internal ribosome entry sites (IRES), N6-methyladenosine (m6A) internal ribosome entry sites (MIRES), and rolling circle amplification (RCA) [61]. Proteins translated from circRNAs, like circ-ZNF609, circPINT (producing PINT87aa), and circSHPRH (producing SHPRH-146aa), exhibit functions distinct from their linear mRNA counterparts. These proteins may regulate cell proliferation or protect native proteins from degradation [62, 63]. The m6A modification plays a critical role. It recruits initiation factors such as eIF4G2 and YTHDF3, even under stress conditions [64, 65]. RCA allows continuous translation from circRNAs with long open reading frames (ORFs), as seen with circEGFR in glioblastoma [66].

#### Clinical implications of circular RNAs

CircRNAs have emerged as significant players in growth, development, and various biological processes, as well as in the context of numerous illnesses [67, 68]. They have been found to be prevalent and persistent in exosomes, detectable in blood and urine [69]. Exosomal circRNAs, which can be transferred across cells, serve a range of functions, including enhancing inflammatory responses, modulating immunity, and regulating malignant cell proliferation, invasion, metastasis, and treatment resistance [70, 71]. Importantly, numerous studies suggest that circRNAs could serve as valuable clinical signatures for early diagnosis and prognosis, as well as promising therapeutic targets [68, 72].

Dysregulated circRNAs, such as circ_014924 and circHECTD1, have been linked to lung inflammation and silicosis [73–75], while others, like circLPAR1 and circCDYL, are associated with cancer detection and progression [76, 77]. Exosomal circRNAs, including circIARS and circRPN2, also play a role in predicting metastasis and patient survival [78]. These findings underscore the clinical potential of circRNAs in early diagnosis and outcome prediction.

#### The potential of circRNAs in targeted therapy

CircRNAs, with their remarkable stability, present a promising avenue for targeted therapy [33]. They can be effectively delivered to target tissues via nanoparticles, exosomes, or viral vectors [79, 80]. For instance, circUBE2D2, upregulated in exosomes from tamoxifen-resistant breast cancer cells, promotes drug resistance by sponging miR-200a-3p. Targeting this circRNA may offer a novel approach to overcome tamoxifen resistance. Furthermore, circRNAs are being investigated as a strategy to combat drug resistance in cancer and as innovative platforms for SARS-CoV-2 vaccines [81, 82].

In conclusion, circRNAs represent a unique class of noncoding RNAs with remarkably diverse biological roles and significant clinical potential. Their covalently closed structure, formed through back-splicing, ensures exceptional stability, thereby making them promising biomarkers and therapeutic targets. CircRNAs regulate gene expression by acting as miRNA sponges, mediating protein interactions, and influencing chromatin remodeling and transcription. Some circRNAs also encode proteins, thereby expanding their functional repertoire. Their involvement in cancer, inflammation, and other diseases highlights their diagnostic and prognostic value. Particularly, exosomal circRNAs detectable in bodily fluids reinforce this potential. Furthermore, their stability and versatility enable innovative therapeutic strategies, such as targeted delivery via nanoparticles or exosomes, to overcome drug resistance. As research advances, circRNAs are poised to transform precision medicine, offering novel solutions for the early diagnosis, prognosis, and treatment of complex diseases, including cancer and infectious diseases, in a rapid manner.

### Role of the Wnt signaling pathway in BC

The Wnt signaling pathway, a complex network of secreted lipoglycoproteins, encoded by 19 distinct Wnt genes in the human genome, plays a crucial role in cellular functions during embryonic development through interactions with diverse receptors [83]. However, when this system is disrupted, it significantly contributes to BC pathogenesis—promoting proliferation, metastasis, progression, and therapeutic resistance. Notably, studies have demonstrated that inhibition of Wnt/β-catenin activity in normal breast tissue results in developmental defects and decreased cellular proliferation, underscoring its fundamental role in breast homeostasis [84]. The Wnt signaling cascade operates through two distinct mechanisms: the β-catenin-dependent (canonical) and β-catenin-independent (noncanonical) pathways. The noncanonical pathway further subdivides into two distinct branches based on specific Wnt ligand–receptor interactions: the planar cell polarity (PCP) pathway and the calcium (Ca2 +) signaling pathway [85].

This section comprehensively examines the Wnt canonical pathway, noncanonical pathways and their specific branches, the planar cell polarity (PCP) pathway, and Wnt/Ca2 + signaling. This detailed exploration will provide a deeper understanding of these pathways and their role in cellular functions and BC.

#### Wnt canonical pathway

The canonical Wnt pathway (β-catenin-dependent) plays a pivotal role in breast tumorigenesis and progression, particularly in identifying neoplastic angioinvasions [86]. This pathway's primary regulatory mechanism centers on precisely controlling intracellular β-catenin levels. In the absence of Wnt proteins, the 'destruction complex'—comprising two key kinases, casein kinase 1α (CK1α) and glycogen synthase kinase three beta (GSK-3β), along with two scaffold proteins, axis inhibition (Axin) and adenomatous polyposis coli (APC)—maintains low intracellular β-catenin levels. The sequential phosphorylation process begins with CK1α targeting serine 45 (Ser45), followed by GSK-3β phosphorylating Ser33, Ser37, and threonine 41 (Thr41), ultimately leading to β-catenin ubiquitination and degradation through beta-transducin repeat-containing protein (βTrCP)[87]. Understanding the intricacies of this pathway is of utmost importance in the fight against BC.

The canonical Wnt signaling requires coordinated action of both Frizzled (FZD) family receptors and low-density lipoprotein-related protein 5/6 (LRP5/LRP6) co-receptors, with receptor activation dependent on LRP5/LRP6 phosphorylation. Upon Wnt binding, Disheveled (DVL) phosphorylation triggers Axin dephosphorylation and decreased cytoplasmic content, releasing β-catenin from the "destruction complex" and enabling its stabilization and nuclear translocation. While multiple nuclear β-catenin binding partners participate in gene transcription regulation, the T-cell factor/lymphoid enhancer factor (TCF/LEF) family of transcription factors represents the most significant interaction partners [88]. The Wnt/β-catenin pathway orchestrates multiple cancer-promoting processes: inhibiting apoptosis, enhancing DNA damage repair, stimulating proliferation, promoting metastasis, inducing epithelial–mesenchymal transition (EMT), and upregulating matrix metalloproteinase (MMP) expression—all contributing to therapeutic resistance and tumor microenvironment remodeling [89].

The canonical Wnt signaling pathway represents a sophisticated molecular cascade that regulates β-catenin levels through complex protein interactions and phosphorylation events, ultimately controlling gene expression patterns crucial for cell fate decisions. This pathway's dysregulation in BC leads to multiple oncogenic processes, including enhanced cell survival, proliferation, and metastatic potential, making it a critical target for therapeutic intervention. Understanding and targeting the Wnt pathway offers hope for effective cancer treatments.

#### Noncanonical pathways

The noncanonical Wnt signaling pathway, a complex system that rivals its canonical counterpart in significance, is emerging as a key player in cancer progression [90]. Research by Remo et al. [86] has unveiled a crucial aspect of this complexity, identifying the nuclear factor of activated T cells 5 (NFAT5) as a significant prognostic indicator in breast carcinoma, particularly in cases resembling inflammatory breast carcinoma.

#### PCP pathway

PCP pathway primarily regulates directional cell movement and cellular morphology through a complex signaling cascade. This pathway is initiated when Wnt ligands, which are secreted glycoproteins that act as signaling molecules, bind to the retinoic acid receptor-related orphan receptor (ROR)-FZD receptor complex, triggering DVL recruitment and activation. Subsequently, DVL interacts with Rho GTPase, leading to coordinated activation of Rac1 and Rho, which in turn activate Rho-associated protein kinase (ROCK) and Jun N-terminal kinase (JNK) to modulate cytoskeletal arrangements and transcriptional responses [91]. Receptor expression patterns exhibit distinct variations across BC subtypes, creating unique molecular signatures. These patterns include elevated Fzd2 in metastatic breast cancer, increased ORAI1 in basal-like breast cancer (BLBC), high expression of Vangl2 and ROR1 in BLBC, and overexpression of Syndecan in BC generally. Conversely, specific receptors show reduced expression, with RYK decreased in primary BC and Glypican silenced across BC cases [25].

The noncanonical Wnt signaling pathways, particularly the PCP pathway, are the conductors orchestrating crucial cellular processes. These complex receptor-mediated mechanisms significantly impact BC progression, providing valuable insights for targeted therapeutic approaches and prognostic evaluation.

#### Wnt/ Ca^2+^

The Wnt/Ca2 + signaling pathway, a complex and intriguing system, emerges as a critical regulator of cell fate decisions and migration, with mounting evidence supporting its significance [92]. This pathway orchestrates calcium release from intracellular stores through a complex signaling cascade [93]. The signaling sequence initiates when Wnt ligands bind to FZD receptors, triggering phospholipase C (PLC) activation, which catalyzes the breakdown of phosphatidylinositol (4,5)-biphosphates (PIP2) into inositol (1,4,5)-triphosphates (IP3) and diacylglycerol (DAG). Through parallel mechanisms, DAG activates protein kinase C (PKC) and subsequently the small GTPase cell division cycle 42 (CDC42), while IP3 stimulates intracellular Ca2 + release. This calcium flux activates both Ca2 + /calmodulin-dependent kinase II (CaMKII) and calcineurin (CaN), leading to downstream effects: CaMKII phosphorylates transforming growth factor (TGF)-β-activated kinase 1 (TAK1), activating Nemo-like kinase (NLK) and inhibiting canonical Wnt signaling, while CaN dephosphorylation activates the nuclear factor of activated T-cells (NFAT), facilitating its nuclear translocation and gene regulation. These coordinated events ultimately control cell motility and cytoskeletal organization [94, 95].

In BC cells, the Wnt signaling pathway components undergo multiple alterations across various molecular levels, underscoring the urgency and importance of understanding its role. These alterations include genetic modifications (mutations, amplifications, deletions, and methylations), posttranscriptional modifications of mRNA, and posttranslational protein modifications, including changes in β-catenin subcellular localization. While mutations in Catenin Beta 1 (CTNNB1), which encodes β-Catenin, are rare in BC [96], Wnt pathway activation remains a fundamental driver of BC development [97].

The Wnt/Ca2 + signaling pathway, a sophisticated molecular network, represents a promising avenue for therapeutic intervention. It regulates cellular behavior through calcium-dependent mechanisms and intricate protein interactions, culminating in critical cell motility and organization changes. The pathway's components undergo complex modifications in BC, highlighting its significance as a therapeutic target despite the relative rarity of direct CTNNB1 mutations. This potential for therapeutic intervention offers hope in the fight against BC).

### Role of CircRNAs in the pathogenesis of BC

Hormones and intricate signaling pathways regulate normal human development, including the proliferation of mammary cells, and facilitate communication between them [98]. Moreover, cancer cells have been seen to dysregulate some of these signaling pathways [99]. The local and remote tumor microenvironments are regulated by epigenetic and genetic modifications, which are vital in avoiding apoptosis, angiogenesis, aging resistance, metastasis, and tumor invasion. The Wnt/β-catenin signaling network has been shown to regulate several biological processes in lung cancer [100]. It is known that abnormal Notch1 signaling pathway activation disturbs the function of the regulatory system regulated by Notch1, eventually leading to CRC [101, 102]. The Hedgehog (Sonic Hedgehog (Shh)) signaling system has been studied and found to have an oncogenic function in several malignancies. In the specific context of rhabdomyosarcoma, this pathway has a dominant role in the development and advancement of cancer [103]. The Janus kinase/signal transducer and activator of transcription (JAK/STAT) signaling system has been demonstrated to participate in transmitting signals through cell-surface receptors and has also participated in the progression, treatment resistance, and metastasis of cervical cancer [104]. The following part briefly summarizes the four crucial signaling pathways and their role in the development of normal human breast cells and BC.

#### TGF-β signaling pathway

TGF-β serves as a master regulator of crucial biological mechanisms, including cellular proliferation, differentiation, apoptosis, autophagy, angiogenesis, and EMT. The biological actions of TGF-β are mediated through two distinct pathways: the SMAD-dependent pathway and the non-SMAD-dependent pathway. The Smad gene nomenclature originates from the fusion of two evolutionarily conserved proteins: the Caenorhabditis elegans small protein (Sma) [105] and the Drosophila gene known as mothers against decapentaplegic' (Mad) [106]. The SMAD-dependent pathway operates through specific Ser/Thr kinase receptors—TGF-β receptor I (TβR I) and TGF-β receptor II (TβR II)—embedded in the cell membrane. When activated, TβR II and TGF-β work synergistically to enhance TβR I kinase activity and stimulate its phosphorylation. Subsequently, activated TβR I phosphorylates SMAD2 and SMAD3 proteins. These phosphorylated SMADs then complex with SMAD4 (the chaperone protein) and translocate to the nucleus, where they regulate TGF-β target gene expression [107, 108].

In BC, the sex-determining region Y box 4 (SOX4) transcription factor mediates TGF-β signaling to promote EMT, cell proliferation, and metastasis [109]. Moreover, TGF-β stimulates double mouse minute 2 (MDM2) production, leading to p53 destabilization and subsequent EMT and tumor formation [110]. Elevated TGF-β levels correlate with increased microvascular density in BC, indicating poor patient prognosis [109]. An oncogene, circDISP3, was identified to be upregulated in TNBC cells by binding to miR-4638-3p through the TGF-β1 signaling pathway that suppresses the expression of activating transcription factor 3 (ATF3), a gene that is induced by stress and has oncogenic properties. This mechanism enhances the proliferation of cells, invasion, and the creation of bone metastases [111].

TGF-β signaling orchestrates multiple cellular processes through SMAD-dependent and non-SMAD-dependent pathways, with its dysregulation playing a crucial role in BC progression. In BC, particularly TNBC, TGF-β pathway alterations involve multiple molecular players, including SOX4, MDM2, and circDISP3, ultimately promoting tumor growth and metastasis. TGF-β and Wnt signaling pathways interact at several levels. Without TGF-β, Smad3 is phosphorylated by glycogen synthase kinase-3β (GSK-3β), leading to its degradation. TGF-β or bone morphogenetic proteins (BMPs) activate Smads, but GSK-3β can still phosphorylate them, thereby reducing their activity. Wnt signaling inhibits GSK-3β, stabilizing Smads and promoting cooperation between β-catenin and Smad3 in gene transcription [112].

#### PI3K/AKT/mTOR signaling pathway

The phosphoinositide 3-kinases (PI3K), protein kinase B (AKT), and mammalian target of rapamycin (mTOR) pathways constitute an interconnected signaling network that orchestrates critical cellular processes, including proliferation, differentiation, and migration [113]. Notably, targeted suppression of this pathway has been shown to inhibit tumor cell development [114]. PI3Ks, functioning as lipid kinases, regulate essential cellular activities—including cell cycle progression, DNA damage response, and apoptosis [115–117]. These enzymes are categorized into three distinct classes based on their structural composition and functional characteristics [118, 119].

The pathway activation typically initiates through PI3K mutations or overexpression in BC. In this process, PI3K catalyzes the phosphorylation of PIP2 to produce phosphatidylinositol 3,4,5-trisphosphate (PIP3). Two key tumor suppressors—inositol polyphosphate 4-phosphatase type II (INPP4B) and phosphatase and tensin homolog (PTEN)—regulate PIP3 through dephosphorylation. AKT, a Ser/Thr protein kinase, interacts explicitly with PIP3 at the cell membrane, which serves as its docking site [120]. Upon membrane recruitment, AKT undergoes phosphorylation and subsequent activation [121]. PI3K interacts with estrogen, insulin, and notably Wnt/β-catenin signaling, with differing effects across breast cancer subtypes. In PIK3CA-mutant ER + breast cancer, PI3Kα enhances Wnt activation via INPP4B-mediated GSK3β degradation. In contrast, in ER-breast cancer, the inhibition of PI3K/mTOR activates Wnt/β-catenin signaling [122].

In BC, the AKT pathway frequently exhibits hyperactivation due to mutations, reinforcements, and the loss of negative regulators [123]. This activation triggers mTOR phosphorylation and activation—a critical modulator of protein synthesis and cellular growth. Notably, mTOR overactivation, commonly observed in BC, results in unrestricted cell proliferation [124]. Recent studies have identified elevated expression of oncogene circ_103809, derived from exons 13 to 17 of the zinc finger RNA binding protein (ZFR) gene [125], which promotes cell development through the PI3K/AKT signaling pathway in BC [126].

The PI3K/AKT/mTOR signaling cascade represents a complex regulatory network essential for cellular homeostasis, with its dysregulation playing a central role in BC development and progression. The pathway's complexity is further exemplified by the involvement of various molecular players, including tumor suppressors and oncogenes like circ_103809, whose altered expression contributes to BC pathogenesis through enhanced cell proliferation and survival.

#### NF-κB signaling pathway

The nuclear factor kappa B (NF-κB) signaling pathway, a key regulator of immune responses and cell survival, is pivotal in immunological disorders and cancer development. Its dysregulation can lead to chronic inflammation and contribute to the development and progression of various diseases, including cancer [127]. The NF-κB family of transcription factors comprises heterodimers and homodimers formed by five distinct monomers: p50, p52, v-rel avian reticuloendotheliosis viral oncogene homolog (Rel)A, cRel, and RelB. This pathway operates through two distinct mechanisms—the canonical and noncanonical—each activated by specific triggers. The canonical NF-κB pathway responds to various external stimuli associated with immunological response, inflammation, cell proliferation, and differentiation. This pathway's initiation involves the inhibitor of nuclear factor kappa B (IκB) proteins (IκBα, IκBβ, IκBε, or IκBγ) and is activated by tumor necrosis factor-alpha (TNF-α), which responds to growth factors, cytokines, mitogens, UV radiation, and bacteria [128].

TNFα binding to tumor necrosis factor receptor 1 (TNFR1) triggers the recruitment of specific proteins—receptor-interacting Ser/Thr-protein (RIP), TNFR1-associated death domain protein (TRADD), and TNF receptor-associated factor 2 (TRAF2). TRAF2 then recruits the NF-κB essential modulator (NEMO), inhibitory kappa B kinase α (IKKα), and IKKβ complex to TNFR1, leading to IκBβ activation and IκBα phosphorylation [129, 130]. Subsequently, IκBα ubiquitination enables NF-κB release to the cytoplasm and nuclear translocation, where it binds to specific DNA sequences—NF-κB sites—activating IL-6 and chemokine gene production [131, 132].

The noncanonical pathway responds exclusively to growth factors, stress, viruses, lipopolysaccharides [131], and select TNF superfamily receptors, indicating more specialized biological functions [133]. In this pathway, CD40 activation of RelB/NF-κB2 leads to TRAF2-TRAF3 engagement, connecting cellular inhibitor of apoptosis (c-IAP) to NF-κB-inducing kinase (NIK). NIK phosphorylation and reduction by c-IAP activates IKKα and degrades the p100 subunit—encoded by the NF-κB2 gene—releasing p52 and RelB for nuclear translocation and chemokine gene production [131, 132].

The recent studies have identified NF-κB as a critical link between inflammation and cancer, particularly in BC development and hormone therapy resistance [134]. Notably, hsa_circ_0043278—located at chr17 in the transcriptional adaptor 2A (TADA2A) gene [135]—shows reduced expression in BC cell lines. This circular RNA directly interacts with miR-455-3p, encoded by the human collagen type XXVII alpha 1 (COL27A1) gene, modulating the NF-κB signaling pathway to inhibit BC cell proliferation and migration [134].

The NF-κB signaling pathway, a complex regulatory network operating through canonical and noncanonical mechanisms, plays crucial roles in inflammation, immune response, and cancer development. Understanding its intricate molecular interactions, particularly in BC, has revealed potential therapeutic targets. These targets, such as hsa_circ_0043278 and its interaction with miR-455-3p, offer promising avenues for the development of novel cancer treatments, instilling hope and optimism in the field of oncology. β-catenin inhibits NF-κB by promoting IκB expression, sequestering NF-κB in the cytoplasm, downregulating TLR4, recruiting transcriptional corepressors, and blocking CBP-mediated acetylation. It suppresses NF-κB-driven gene expression at multiple levels. Conversely, β-catenin can activate NF-κB by inducing p38 MAPK signaling. It also stabilizes BTRC (βTrCP) mRNA via Coding region determinant-binding protein (CRD-BP), enhancing IκB degradation and NF-κB activation. Additionally, it upregulates TNFRSF19 and cooperates with NF-κB at the transcriptional level. The interplay between the Wnt/β-catenin and NF-κB signaling pathways presents new opportunities for treating inflammatory and inflammation-related diseases, including cancer [136].

#### MAPK signaling pathway

The mitogen-activated protein kinases (MAPKs) constitute a comprehensive network of Ser/Thr kinases that initiate phosphorylation cascades, transmitting signals from the cellular surface to the nucleus [137]. Within this pathway, the Rat sarcoma (RAS) protein superfamily—comprising guanosine diphosphate (GDP) and guanosine triphosphate (GTP)-binding GTPase—plays a fundamental role in signal transduction. The MAPK signaling cascade initiates when epidermal growth factor receptor (EGFR) stimulation activates RAS GTPase, which catalyzes GTP hydrolysis to GDP and inorganic phosphate [138].

RAF, functioning as a downstream mediator of RAS, requires direct interaction with activated RAS proteins. RAF kinases, a specialized class of protein kinases targeting Ser/Thr residues, serve as critical pathway modulators by activating the MAPK kinase kinase (MAPKKK). This activation triggers a sequential phosphorylation cascade through MAPK kinase (MAPKK: MEK1/2/3/4/5/6/7) and culminates in MAPK activation. The MAPK family encompasses three primary kinases with distinct isoforms: p38 MAPKs, extracellular signal-regulated kinases (ERKs), and JNKs. In normal cellular processes, the ERK/MAPK signaling pathway orchestrates crucial physiological processes, including cell proliferation, differentiation, apoptosis, and tissue development. However, when this pathway is dysregulated, it can contribute to tumorigenesis [139, 140]. The ERK/MAPK signaling pathway orchestrates crucial physiological processes—including cell proliferation, differentiation, apoptosis, and tissue development—while contributing to tumorigenesis [141]. Notably, elevated ERK expression has been observed across multiple human malignancies, particularly in BC [142]. The recent studies have identified circ_0006528, derived from exons 2 to 5 of the PRELID gene, as a key regulator that targets miR-7-5p through the ERK/MAPK signaling pathway and RAF1 activation, promoting BC cell proliferation, migration, and invasion. Circ_0006528 plays a significant role in the dysregulation of the ERK/MAPK signaling pathway, thereby contributing to the progression of BC [143].

The MAPK signaling pathway represents a sophisticated cellular communication system that regulates crucial biological processes through sequential kinase activation cascades. Its 'dysregulation ', which refers to the abnormal or uncontrolled activation of the pathway, is implicated in various cancers. In BC specifically, the pathway's complexity is exemplified by the interaction between circular RNA circ_0006528 and miR-7-5p, which influences tumor progression through ERK/MAPK signaling and RAF1 activation. The scaffold proteins Axin and APC, as well as the kinase GSK3, indicate that the destruction complex in colon cancer is inhibited by stimulation of the Wnt/β-catenin signaling pathway. This results in Ras stabilization, which in turn activates the downstream MAPK signaling cascade. Conversely, in melanoma, elevated MAPK signaling stabilizes Axin, which in turn inhibits Wnt signaling. This highlights bidirectional regulation between the two pathways in cancer [144].

In conclusion, the intricate interplay between various signaling pathways—TGF-β, PI3K/AKT/mTOR, NF-κB, and MAPK—plays a crucial role in BC pathogenesis. These pathways regulate essential cellular processes, including proliferation, differentiation, apoptosis, and metastasis. Recent research has revealed the significant involvement of circRNAs in modulating these pathways, with specific molecules such as circDISP3, circ_103809, hsa_circ_0043278, and circ_0006528 emerging as key regulators. Their interactions with various microRNAs and downstream effectors contribute to BC development, progression, and therapeutic resistance. Understanding these complex molecular mechanisms and pathway interactions is of utmost importance, as it provides promising opportunities for developing targeted therapeutic strategies in BC treatment. The profiles of circRNA expression and associated pathways in BC are outlined in Table 1.

**Table 1:** Expression profile of circRNAs and related pathways in BC

Author & Year	Function	Circ RNA	Target	Pathway	Effect	Sample study
Zhao et al. 2023 [199]	Oncogene	Circ_0008784	miR-506–3p/CTNNB	Wnt/β-catenin	Promoted proliferation and inhibited apoptosis	BC, TNBC, kidney cells
Li et al. 2022 [200]	Oncogene	CircEIF6	EIF6-224 aa/MYH9	Wnt/β-catenin	Promoted metastasis and proliferation of TNBC cells	BC, nude mice
Zhu et al. 2021 [178]	Oncogene	CircDONSON	SOX4	Wnt/β-catenin	Promoted cell proliferation and radioresistance	BC, breast epithelial cells
Leng et al. 2021 [201]	Oncogene	Circ_0000043	miR-136	TGF-β/Smad3	Promoted EMT, migration, proliferation, and invasion	BC, breast epithelial cells
Zeng et al. 2018 [202]	Oncogene	CircANKS1B	miR-148a/152-3p/USF1	TGF-β1/Smad	Promoted invasion and metastasis	BC, nude mice
Zhang et al. 2024 [203]	Oncogene	CircRPPH1	miR-326/ITGA5/FAK	PI3K/AKT	Promoted proliferation, migration, and invasion	BC, TNBC, HEK293T cells, nude mice
Ju et al. 2023 [204]	Oncogene	Circ_0042881	miR-217/SOS1/RAS	PI3K/AKT	Promoted invasion, migration, and proliferation	BC, mammary cells, mice
Wang et al. 2022 [205]	Oncogene	CircUBAP2	miR-300/ASF1B	PI3K/AKT/mTOR	Increased resistance of TNBC to cisplatin	TNBC, breast epithelial cells
Wang et al. 2022 [206]	Oncosuppressor	CircSEMA4B	miR-330-3p/PDCD4	PI3K/AKT	Inhibited proliferation and migration	BC, breast epithelial cells, nude mice
Zhou et al. 2022 [207]	Oncogene	Circ_0006014	miR-885-3p/NTRK2	PIK3CA/AKT	Induced invasion, migration, and proliferation	BC, nude mice
Liu et al. 2022 [208]	Oncosuppressor	CircRNF10	miR-934/PTEN	PI3K/AKT	Repressed proliferation, invasion, and migration; enhanced NK cell effectiveness	BC cell lines, mice
Li et al. 2021 [209]	Oncogene	Hsa_circ_0000199	miR-206/613-led	PI3K/Akt/mTOR	Promoted proliferation, invasion, migration, and chemotherapy resistance	BC, mammary epithelial cells, nude mice
Chen et al. 2020 [210]	Oncogene	CirCHIPK3	miR‐193a/HMGB1	PI3K/AKT	Promoted invasion, migration, and proliferation	BC, nude mice
Zhou et al. 2023 [211]	Oncogene	CircSERPINE2	MALT1/IL-6	NF-κB	Promoted proliferation and invasion	BC, mice
Zheng et al. 2023 [212]	Oncosuppressor	CircRNF10	DHX15/p65	NF-κB	Repressed migration and proliferation	BC, HEK293T cells, nude mice
Wang et al. 2021 [213]	Oncogene	CircTPGS2	miR-7/TRAF6	NF-κB	Promoted inflammatory-promoting chemokine construction, migration, and induced tumor-associated inflammation	BC, nude mice
Xu et al. 2021 [214]	Oncogene	CircIKBKB	M-CSF and GM-CSF	NF-κB	Promoted osteoclastogenesis and BC-bone metastasis by causing bone pre-metastatic niche formation	BC, nude mice
Jiang et al. 2020 [166]	Oncogene	CircABCC4	miR-154-5p	NF-κB	Promoted proliferation, invasion, and migration	BC
Lee et al. 2023 [215]	Oncosuppressor	CircAAGAB	miR-378h/p38	MAPK	Inhibited proliferation and migration; increased radiosensitivity	BC, Breast epithelial cells
Wang et al. 2023 [216]	Oncogene	CircDNAJC11	TAF15	MAPK	Promoted invasion, proliferation, and migration	BC, nude mice
Gao et al. 2019 [143]	Oncogene	Hsa_circ_0006528	miR-7-5p/Raf1	MAPK/ERK	Promoted proliferation, migration, and invasion	BC, breast epithelial cells
Xu et al. 2023 [217]	Oncogene	CircAR-E2E4	miR-665	STAT3	Promoted proliferation of TNBC cells	TNBC, nude mice
Wang et al. 2021 [218]	Oncosuppressor	CircNOL10	miR-767-5p/SOCS2	JAK2/STAT5	Suppressed invasion and proliferation; induced apoptosis	BC, breast epithelial cells, nude mice
Zhang et al. 2021 [219]	Oncogene	CircRHOT1	miR-106a-5p	STAT3	Promoted invasion, proliferation, and migration; impeded apoptosis	BC, nude mice
Fu et al. 2021 [220]	Oncogene	CircBCBM1	miR-125a/BRD4	SHH/MMP9	Promoted proliferation, metastasis, and migration	Brain-targeting BC cells, nude mice
Ye et al. 2020 [221]	Oncogene	CircDCAF6	miR-616-3p	GLI1-SHH	Promoted proliferation	BC, breast epithelial cells
Yao et al. 2021 [173]	Oncogene	CircFAT1	miR-525-5p/SKA1	Notch	Promoted proliferation and migration	BC, breast epithelial cells
Wang et al. 2018 [222]	Oncosuppressor	CircRNA-000911	miR‑449a	Notch1	Suppressed proliferation, migration, and invasion; triggered apoptosis	BC, breast epithelial cells

*BC* BC cell lines, breast epithelial cells: normal breast epithelial cells

### Molecular mechanisms of circRNA regulation in breast cancer: insights into regulatory roles

The majority of circRNAs, even without numerous protein-binding sites, serve as protein decoys or sponges with the capacity to bind a single protein effectively. This binding inhibits the recruitment of proteins, thereby modulating downstream molecular transcription or preventing specific proteins from interacting with downstream molecules [145, 146]. For instance, circACTN4, which is upregulated in BC cases and associated with poor prognosis, metastasis, and advanced clinical stage [147], directly binds to Far Upstream Element Binding Protein 1 (FUBP1) in BC. FUBP1 regulates RNA biogenesis, DNA transcription, and translation through its interactions with RNA and DNA. The interaction between circACTN4 and FUBP1 promotes the transcription of the MYC protein, thus enhancing cancer progression [148, 149]. Furthermore, MYC, a multifunctional transcription factor, influences critical processes, including DNA damage response, apoptosis, and cell cycle regulation [150].

Additionally, circ_0068631 is upregulated in BC cell lines and tissues. It has been established that circ_0068631 is associated with distant or lymph node metastasis, tumor size, and advanced cancer stage [151]. Notably, hsa_circ_0068631 binds to EIF4A3 (eukaryotic translation initiation factor 4A3). EIF4A3 exhibits significant activity in mRNA degradation, translation, splicing, and transport [152–154]. On the other hand, circRNAs also serve as protein scaffolds, either inhibiting or facilitating interactions between proteins [155, 156]. CircFoxo3 regulates stress resistance, cell apoptosis, and cell cycle progression. Research indicates that its expression is reduced in BC. CircFoxo3 binds to the ubiquitin enzyme and p53 protein, thereby promoting p53 ubiquitination and degradation. Consequently, this interaction suppresses BC growth and induces apoptosis [157].

RNA-binding proteins (RBPs) regulate RNA degradation, splicing, and transcription by binding to RNA-binding domains [158, 159]. CircRNA–RBP interactions play a critical role in BC pathogenesis [151, 160]. For instance, circ-1073, through specific mechanisms, functions as a tumor suppressor in BC and promotes cell apoptosis (Citation88). CircRNAs also modulate transcription in BC by interacting with RNA polymerase II. CircScrib570 inhibits pre-mRNA splicing and translation, upregulates E-cadherin, and downregulates N-cadherin and vimentin, thereby promoting breast cancer proliferation and invasion. Similarly, CircACTN4 upregulates MYC transcription, enhances CDK4 and CCND2 expression, and drives breast cancer development and metastasis through MYC activation [161].

To sum up, circRNAs play a pivotal role in breast cancer progression by mediating protein-RNA interactions and post-transcriptional modifications. Functioning as protein sponges or scaffolds, circRNAs such as circACTN4, circ_0068631, and circFoxo3 regulate MYC, EIF4A3, and p53 activities, thereby modulating proliferation, metastasis, and apoptosis. Robust experimental evidence, including RNA pull-down assays and knockout studies, substantiates these roles. A deeper understanding of these molecular mechanisms, particularly protein–RNA interactions and RNA modifications, underscores circRNAs as promising therapeutic targets for effective breast cancer management.

### Intricate interplay of CircRNA and Wnt signaling pathways in BC

circRNA significantly influences BC development and pathophysiology. Wang et al. demonstrated that circRNAs modulate multiple critical signaling cascades, including TGF-β, Wnt, erythroblastic leukemia viral oncogene homologue (ErbB), p53, and microRNA pathways in cancer [162]. This section delves into the potential of key circRNAs and their regulatory mechanisms through interaction with Wnt signaling pathways.

#### CircABCC4

CircABCC4, a multipathway oncogenic regulator, functions across a spectrum of cancers, including prostate cancer [163], lung adenocarcinoma [164], and bronchopulmonary dysplasia [165], with particular significance in BC [166]. The recent research from 2020 revealed elevated circABCC4 levels in BC patient tissues. In breast tumor cell lines, circABCC4 downregulation suppressed proliferation, migration, and metastasis while promoting apoptosis. CircABCC4 exhibited inhibitory effects on miR-154-5p activity at corresponding binding sites. Notably, miR-154-5p suppression partially reversed the inhibitory effects of circABCC4 reduction on BC cell survival, motility, metastasis, and the NF-κB and Wnt/β-catenin pathways [6]. These findings align with the known roles of specific nuclear factor-κB complexes in T-cell regulatory development [167]. Through miR-154-5p upregulation, circABCC4 reduction inhibited BC cell growth, movement, and invasion by suppressing the NF-κB and Wnt/β-catenin signaling pathways [166].

#### CircFAT1

CircFAT1 serves as an oncogenic regulator in multiple conditions, including lung adenocarcinoma progression [168], cervical cancer malignant progression [169], invasion of malignant melanoma [170], diabetic retinopathy [171], suppression of CRC development [172], and BC [173]. Yao et al. discovered elevated circFAT1 activation in oxaliplatin (OX)-resistant BC. CircFAT1 reduction in OX-resistant BC cells significantly enhanced apoptosis while reducing OX IC50 values, cell motility, and invasion capacity. Mechanistically, circFAT1 confers OX resistance through inverse regulation of miR-525-5p in OX-resistant BC cells. The circFAT1/miR-525-5p/SKA1 (spindle and kinetochore-associated complex subunit 1) axis promotes metastasis and reduces apoptosis in OX-resistant BC cells. Importantly, SKA1 reduction in BT474/OX cells decreased β-catenin, Notch2, and GSK-3β levels, suggesting SKA1's potential role in activating Notch and/or Wnt signaling pathways [173].

#### CircARL8B

Recent research has demonstrated that silencing the oncogenic regulator circARL8B significantly inhibits BC growth through multiple mechanisms. Wu et al. revealed substantial circARL8B expression in BC tissues. In vitro studies showed that circARL8B knockdown significantly impairs BC cells' proliferative, migratory, and invasive capabilities while also disrupting fatty acid metabolism. These effects were further validated in vivo, where circARL8B silencing prevented tumor formation. Mechanistically, circARL8B functions through targeted suppression of miR-653-5p in BC cells, leading to decreased miR-653-5p levels. The inhibitory effects of circARL8B silencing on BC growth were attenuated when miR-653-5p was reduced, confirming miR-653-5p as a critical downstream target. Further investigation revealed that miR-653-5p inhibits cell survival, motility, metastasis, and fatty acid utilization through interaction with HMGA2 (High Mobility Group AT-Hook 2) in BC cells. This finding is particularly significant given that HMGA2 dysregulation is a hallmark of BC [174], which influences gene activity through chromatin maintenance and DNA minor groove binding [175].

Notably, circARL8B silencing also inhibits the PGE2/PI3K/AKT/GSK-3β/Wnt/β-catenin pathway, ultimately suppressing BC cell survival, movement, metastasis, and fatty acid metabolism through the miR-653-5p/HMGA2 axis [176].

#### CircDONSON

CircDONSON, an oncogenic regulator previously implicated in gastric cancer and glioma development [14, 177, 178], has emerged as a crucial mediator of breast tumor growth through the Wnt/β-catenin pathway modulation. Zhu et al. demonstrated significant upregulation of circDONSON in BC cells and cell lines. Their research revealed that circDONSON reduction decreases BC cell radioresistance while suppressing SOX4 expression and inhibiting BC growth. The mechanism involves circDONSON's activation of Wnt/β-catenin signaling through SOX4—a transcription factor within the group C SOX subclass that plays essential roles in tissue formation during embryogenesis [178, 179].

CircDONSON reduction decreased Wnt1 and β-catenin protein expression in BC cells; notably, SOX4 upregulation reversed these effects. These findings establish that circDONSON activates the Wnt/β-catenin pathway in BC cells through SOX4-mediated signaling, ultimately demonstrating that circDONSON reduction suppresses development and reduces radio-resistance in BC cells through modulation of both Wnt/β-catenin and SOX4 signaling pathways [178].

#### CircITCH

Circular RNA ITCH (circITCH), recognized for its tumor-suppressive properties, has been linked to the etiology of several types of cancer [180], such as bladder cancer [181], melanoma [182], liver [183], thyroid cancer [184] and triple-negative breast cancer [185]. The existing research has indicated that circITCH functions as a Wnt pathway negative regulator, leading to a reduction in tumorigenic potential in various cancers [183, 184, 186] Circ-ITCH was found to be reduced in triple-negative breast cancer by Wang et al. Subsequent research showed that cicrc-ITCH could attenuate the Wnt/β-catenin signaling pathway by functioning as a molecular sponge for miR-214 and miR-17. Elevated levels of the ITCH protein were shown to stimulate ubiquitin ligase function and phosphorylated Dvl breakdown. This inhibition extended to downstream genes, including Axin, c-Myc, and cyclin D1, ultimately weakening the spread, invasive, and migratory capabilities of BC cells [185].

In summary, this section highlights the complex interplay between circular RNAs and the Wnt/β-catenin signaling pathway in BC progression. The discussed circRNAs (circABCC4, circFAT1, circARL8B, circDONSON, and circITCH) demonstrate diverse regulatory mechanisms, primarily through microRNA interactions and downstream effectors. These molecules play a pivotal role in influencing crucial processes, including cell proliferation, metastasis, drug resistance, and metabolism. Notably, circITCH, a key player, acts as a tumor suppressor by inhibiting Wnt/β-catenin signaling through the sponging of miR-214 and miR-17, thereby reducing BC cell invasion and migration. Understanding these intricate molecular networks is of paramount importance as it provides valuable insights into potential therapeutic targets for breast cancer treatment, particularly in addressing challenges such as drug resistance and tumor progression. Additional examples are included in Table 2.

**Table 2:** The intricate interplay of circRNA and Wnt signaling pathway

Author. Year	Function	CircRNA	Target	Effect	Effect on Wnt signaling pathway	Sample study
Jiang et al. 2020 [166]	Oncogenic	CircABCC4	miR-154-5p	Reduced viability and migration; enhanced apoptosis	Knockdown of CircABCC4 led to the NF-κB and Wnt/β-catenin inhibition by increasing miR-154-5p expression	BC, breast adenocarcinoma cells
Zhao et al. 2023 [199]	Oncogenic	Circ_0008784	miR-506-3p/CTNNB1 axis	Promoted apoptosis and proliferation	Circ_0008784 promoted the Wnt/β-catenin activation	BC epithelial cells, TNBC, kidney cells
Zhang et al. 2019 [24]	Oncogenic	CircRNA_069718	β-catenin, c-Myc, and cyclin D1	Inhibited invasion and proliferation	Suppression of circRNA_069718 downregulated β-catenin expression at the transcriptional and translational levels in breast cancer (BC) cells	BC, TNBC, mammary epithelial cells (adenocarcinoma, ductal carcinoma)
Wang et al. 2019 [185]	Oncosuppressor	CircITCH	miR-214/miR-17/ITCH	Inhibited proliferation, invasion, and metastasis	By sequestering miR-214 and miR-17, circ-ITCH operates as a molecular absorbent to suppress Wnt/β-catenin cascade activation in TNBC	BC tissue, breast cells (adenocarcinoma, ductal carcinoma)
Zhu et al. 2021 [178]	Oncogenic	CircDONSON	SOX4	Inhibited proliferation and radioresistance	circ-DONSON promotes Wnt/β-catenin pathway activation in BC cells through SOX4	BC tissue, epithelial cells (fibrocystic, adenocarcinoma, ductal)
Yao et al. 2021 [173]	Oncogenic	CircFAT1	miR-525-5p/SKA1	Promoted apoptosis; inhibited migration, invasion, and drug resistance	Through competitive binding of miR-525-5p, circFAT1 upregulates SKA1 expression, which subsequently modulates the activity of both Notch and Wnt signaling cascades	BC tissue, ductal carcinoma cells
Wu et al. 2021 [176]	Oncogenic	CircARL8B	miR-653-5p/HMGA2, PGE2/PI3K/AKT/GSK-3β	Inhibited viability, migration, invasion, and metabolism; suppressed tumor growth	circARL8B silencing potentially inhibits the PGE2-mediated PI3K/AKT/GSK-3β cascade, thereby suppressing Wnt/β-catenin signaling cascades	BC tissue, fibrocystic cells
Li et al. 2022 [200]	Oncogenic	CircEIF6	EIF6-224aa/ MYH9	Inhibited growth, migration, and invasion	The EIF6-224aa peptide encoded by circ-EIF6 enhances MYH9 protein stability by inhibiting its ubiquitin-mediated degradation, leading to Wnt/β-catenin pathway activation and oncogenic effects	BC tissue, adenocarcinoma cells
Yang et al. 2021 [223]	Oncogenic	circPSMA1	miR-637/Akt1-β-catenin	Promoted tumorigenesis, metastasis, and immunosuppression	circPSMA1 may enhance the levels of Akt1, cyclin D1, and β-catenin, key regulators of cell proliferation and survival pathways	TNBC tissue, BC cell line

### Clinical applications of circular RNAs in breast cancer management

Concerning chemotherapy resistance in BC, dysregulated circRNAs have been identified, suggesting their potential to either mitigate or exacerbate resistance to chemotherapy. CircRNAs associated with drug resistance represent promising therapeutic and diagnostic biomarkers. By enhancing chemotherapy sensitivity, these biomarkers may improve clinical outcomes. Furthermore, in resistant cases, circRNAs facilitate the assessment of treatment efficacy with chemotherapeutic agents. The medications most commonly associated with circRNA-mediated resistance in BC include paclitaxel (PTX), tamoxifen (TAM), and adriamycin (ADM) [187].

For instance, research has demonstrated that in TAM-resistant BC cells, circ_0025202 expression is significantly reduced [52]. Circ_0025202 acts as a sponge for miR-182-5p, suppressing migration, colony formation, and cell proliferation while enhancing tamoxifen sensitivity and promoting apoptosis [188]. Additionally, BC cells exhibiting ADM resistance display marked upregulation of circ_0001667 [189]. Furthermore, circ_0001667 contributes to ADM resistance in BC cells. Its knockdown substantially diminishes growth, migration, invasion, and ADM resistance in malignant cells, indicating its role in promoting drug resistance [190]. The circular RNA Circ_0006528 is overexpressed in PTX-resistant BC tissues and cells, significantly enhancing migration, proliferation, and invasion of PTX-resistant cells, thereby augmenting drug resistance [191].

Assessment of prognostic factors is critical for improving survival outcomes in patients with cancer. Extensive research has established that circRNAs serve diverse roles as prognostic indicators in various BC subtypes [192]. CircRNAs are highly stable molecules, widely prevalent across different pathways, and their expression varies depending on tissue type and cellular stage. These molecules are well-conserved across cell types and resistant to RNaseR degradation [193]. Consequently, circRNAs, owing to their unique metabolic properties, hold potential as cancer biomarkers.

For example, upregulation of circ_0103552 in BC cells is associated with poor prognosis in BC cases [194]. Another prognostic factor, elevated expression of circ_0069094, is linked to adverse outcomes. This circRNA inhibits miR-59, leading to increased glycolysis, which enhances cancer cell aggressiveness and alters the tumor microenvironment [195].

Conversely, certain circRNAs are downregulated in BC and inhibit its growth and progression, suggesting their potential as novel therapeutic agents for BC. For instance, reduced circDDX17 expression in BC cases is frequently associated with diminished long-term survival [196]. Similarly, in BC patients, low circNR3C2 expression is inversely correlated with mortality and the spread of invasive breast cancer [197]. CircNR3C2 serves as a favorable prognostic factor in BC by suppressing migration, invasion, and epithelial–mesenchymal transition progression, while also impeding BC advancement through Vimentin degradation [198].

In summary, circRNAs play a pivotal role in BC management by influencing chemotherapy resistance and prognosis. Dysregulated circRNAs, such as circ_0025202, circ_0001667, and Circ_0006528, modulate resistance to paclitaxel, tamoxifen, and adriamycin, serving as potential biomarkers to enhance treatment sensitivity and assess efficacy. Additionally, circRNAs like circ_0103552 and circ_0069094 correlate with poor prognosis, while circDDX17 and circNR3C2, when downregulated, indicate adverse outcomes, highlighting their therapeutic and diagnostic potential.

## Conclusion

**Fig. 1:**
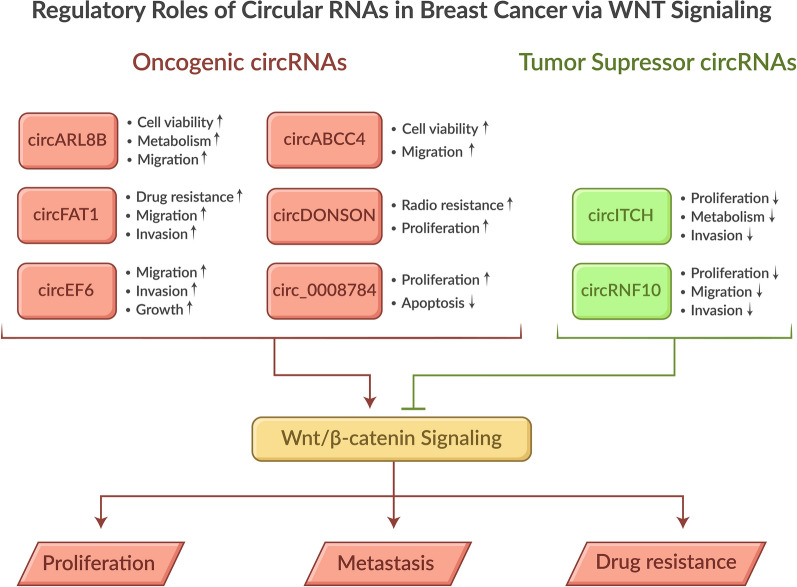
Graphical abstract

circRNAs have emerged as crucial regulators in BC development and progression, mainly through their interaction with various signaling pathways, including Wnt/β-catenin. Several key circRNAs demonstrate significant roles in modulating breast cancer cell behavior, such as circABCC4, circFAT1, circARL8B, and circDONSON. These molecules function primarily through microRNA interactions (acting as miRNA sponges) and influence downstream pathways that control cell proliferation, migration, invasion, and drug-resistance (Fig.1).

Research has revealed that circRNAs can act as either oncogenes or tumor suppressors. For example, circDONSON promotes breast cancer cell proliferation and radioresistance through SOX4-mediated Wnt/β-catenin signaling, while other circRNAs, like circRNF10, exhibit tumor-suppressive properties. The stability and tissue-specific expression patterns of circRNAs make them particularly promising as potential diagnostic biomarkers and therapeutic targets in BC treatment.

**Fig. 2:**
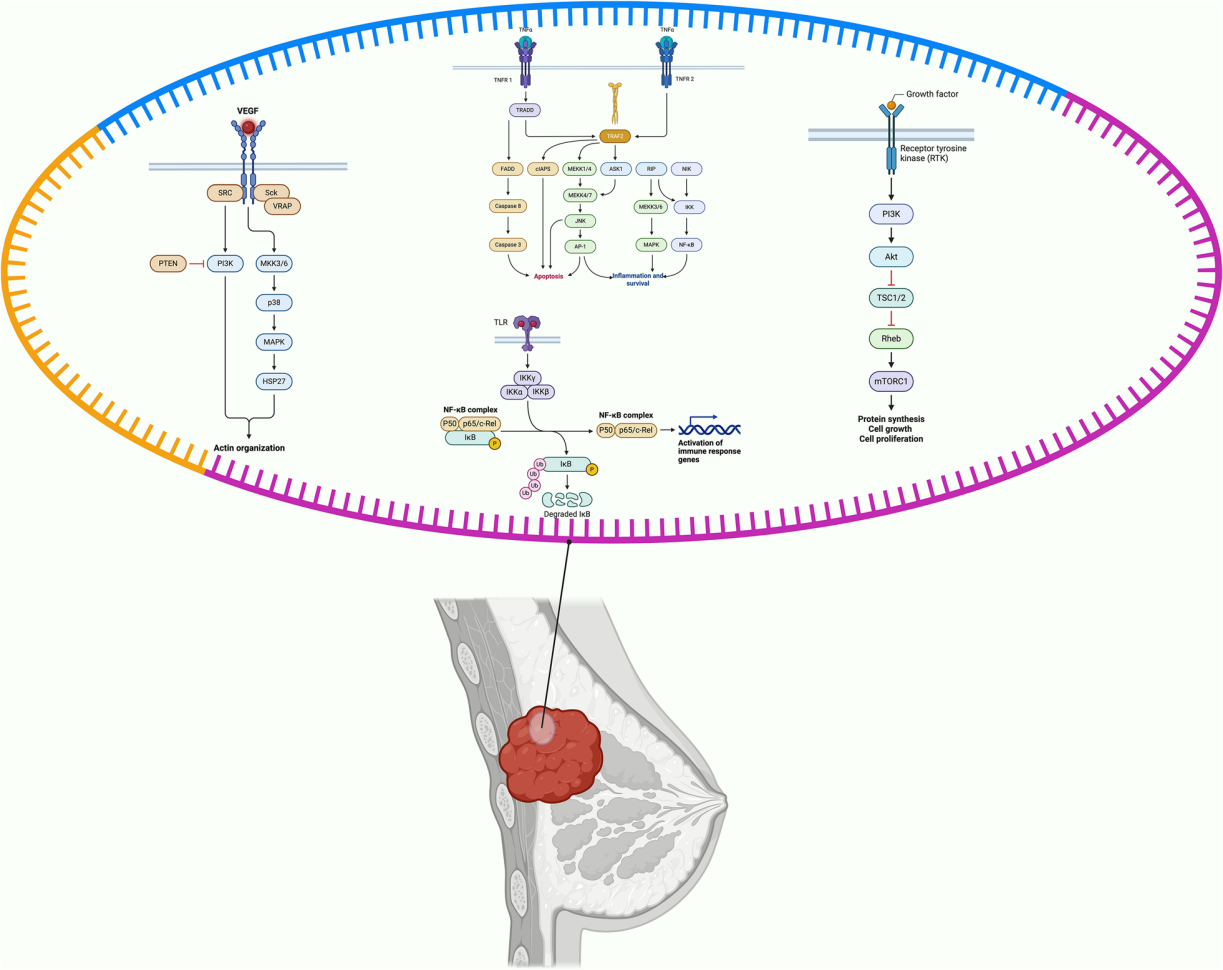
Circular RNAs as modulators of key signaling pathways in breast cancer pathogenesis. Created in BioRender. Khalaji, A. (2025) https://BioRender.com/kb2ulhy

Understanding these complex molecular interactions has opened new avenues for targeted BC therapies. The unique characteristics of circRNAs, including their closed circular structure and resistance to degradation, position them as valuable tools for both diagnostic and therapeutic applications in breast cancer management (Fig.2).

The included studies primarily rely on in vitro and in vivo models, which may constrain their translational relevance to human breast cancer. Few investigations incorporate patient-derived samples or assess clinical outcomes, thereby limiting generalizability to diverse patient populations. Variability in experimental designs, such as differences in cell lines and animal models, coupled with inconsistent circRNA quantification methods, hinders cross-study comparisons. Future research should employ patient-derived xenografts and large-scale clinical cohort studies to enhance clinical applicability. Moreover, standardizing circRNA quantification protocols and validating findings across diverse human populations are essential. Additionally, integrating multi-omics approaches could yield deeper insights into circRNA regulatory mechanisms, thus strengthening the evidence base for therapeutic development.

## Data Availability

No datasets were generated or analysed during the current study.

## Abbreviations

circRNAs: Circular RNAs
BC: Breast cancer
ncRNAs: Non-coding RNAs
piRNA: PIWI-interacting RNA
miRNA: MicroRNA
lncRNA: Long non-coding RNA
CRCs: Colorectal cancers
Wnt: Wingless-related integration site
TNBC: Triple-negative breast cancer
PCP: Planar cell polarity
CK1α: Casein kinase 1α
GSK-3β: Glycogen synthase kinase three beta
APC: Adenomatous polyposis coli
Ser45: Serine 45
Thr41: Threonine 41
βTrCP: Beta-transducin repeat-containing protein
FZD: Frizzled
LRP5/LRP6: Low-density lipoprotein-related protein 5/6
DVL: Disheveled
TCF/LEF: T-cell factor/lymphoid enhancer factor
EMT: Epithelial-mesenchymal transition
MMP: Matrix metalloproteinase
NFAT5: Nuclear factor of activated T cells 5
ROR: Receptor-related orphan receptor
ROCK: Rho-associated protein kinase
JNK: Jun N-terminal kinase
BLBC: Basal-like breast cancer
PLC: Phospholipase C
PIP2: Phosphatidylinositol (4,5)-biphosphates
IP3: Inositol (1,4,5)-triphosphates
DAG: Diacylglycerol
PKC: Protein kinase C
CDC42: Cell division cycle 42
CaMKII: Ca2 + /calmodulin-dependent kinase II
CaN: Calcineurin
TAK1: Transforming growth factor (TGF)-β-activated kinase 1
NLK: Nemo-like kinase
NFAT: Nuclear factor of activated T-cells
CTNNB1: Catenin beta 1
Shh: Sonic Hedgehog
JAK/STAT: Janus kinase/signal transducer and activator of transcription
Sma: Small protein
TβR I: TGF-β receptor I
TβR II: TGF-β receptor II
SOX4: Sex-determining region Y box 4
MDM2: Double mouse minute 2
ATF3: Activating transcription factor 3
PI3K: Phosphoinositide 3-kinases
mTOR: Mammalian target of rapamycin
PIP3: Phosphatidylinositol 3,4,5-trisphosphate
INPP4B: Inositol polyphosphate 4-phosphatase type II
PTEN: Phosphatase and tensin homolog
ZFR: Zinc finger RNA binding protein
NF-κB: Nuclear factor kappa B
TNFR1: Tumor necrosis factor receptor 1
RIP: Specific proteins—receptor-interacting Ser/Thr-protein
TRADD: TNFR1-associated death domain protein
TRAF2: TNF receptor-associated factor 2
NEMO: NF-κB essential modulator
IKKα: Inhibitory kappa B kinase α
NIK: NF-κB-inducing kinase
TADA2A: Transcriptional adaptor 2A
COL27A1: Collagen type XXVII alpha 1
MAPKs: Mitogen-activated protein kinases
RAS: Rat sarcoma
GDP: Guanosine diphosphate
GTP: Guanosine triphosphate
EGFR: Epidermal growth factor receptor
MAPKK: KMAPK kinase kinase
MAPKK: MEK1/2/3/4/5/6/7: MAPK kinase
ERKs: Extracellular signal-regulated kinases
ErbB: Erythroblastic leukemia viral oncogene homologue
OX: Oxaliplatin
SKA1: Spindle and kinetochore-associated complex subunit 1
HMGA2: High Mobility Group AT-Hook 2
